# Transcriptome analysis reveals DNA repair–related clues associated with divergent leaf nuclear DNA diversity in *Leymus chinensis*

**DOI:** 10.3389/fpls.2026.1804463

**Published:** 2026-04-10

**Authors:** Haoyang Yu, Xinxia Wang, Gaowa Naren, Riqing Hao, Huihui Shi, Shumeng Ma, Lingang Zhang

**Affiliations:** 1 School of Life Sciences, Inner Mongolia University/Key Laboratory of Herbage and Endemic Crop Biotechnology, Ministry of Education, Hohhot, Inner Mongolia, China; 2 College of Life Sciences, Inner Mongolia Agricultural University, Hohhot, Inner Mongolia, China

**Keywords:** base excision repair, BRCT domain, intraorganismal genetic heterogeneity, *Leymus chinensis*, mutation

## Abstract

Intraorganismal genetic heterogeneity (IGH) arises from the accumulation of somatic mutations during plant growth. Although leaves of *Leymus chinensis* exhibit pronounced IGH, its molecular basis remains unclear. Under strictly controlled growth conditions, this study compared a wild population (LC-W) with the cultivated cultivar Zhongke No. 2 (LC-ZK2) to search for DNA repair–related clues associated with leaf nuclear DNA diversity. Genomic DNA amplification and Sanger sequencing of three nuclear loci (*MCM7*, *PsaE*, and *PsaL*) showed that, compared with LC-W, LC-ZK2 exhibited fewer polymorphic sites and lower haplotype diversity, indicating a more restricted leaf-scale sequence heterogeneity. *De novo* leaf transcriptome analysis identified 3,833 differentially expressed genes (DEGs; |log_2_FC| ≥ 1, FDR < 0.05; log_2_FC = log_2_[LC-W/LC-ZK2]). GO and KEGG analyses indicated that DEGs were significantly enriched in DNA damage response and DNA repair pathways, with particularly prominent enrichment of base excision repair (BER) and homologous recombination (HR). The BER scaffold gene *XRCC1* plays an important role in these pathways and was significantly upregulated in LC-ZK2 (~2.6-fold), suggesting transcriptional differences in repair-related genes between the two materials. Further Sanger sequencing of the *XRCC1* BRCT domain indicated that LC-ZK2 possessed a more concentrated haplotype spectrum and exhibited distinct amino acid substitution combinations, providing candidate sites for subsequent functional validation. Overall, this study links differences in nuclear DNA diversity with repair-associated transcriptomic signatures and provides an interpretive framework for understanding leaf-scale heterogeneity divergence in *L. chinensis*.

## 1 Introduction

Somatic mutations and the resulting intraorganismal genetic heterogeneity (IGH) are widespread in plants (Schoen and Schultz, 2019). Because plants lack an early, strictly segregated germline, continuous cell division during vegetative growth and meristem differentiation can result in the accumulation of somatic mutations across distinct cell lineages (Lanfear, 2018). In long-lived systems (e.g., oaks), somatic mutation differences have been observed among tissue layers, organs, and branches (Jia et al., 2021; Goel et al., 2024). Somatic cells may also undergo genetic variation processes distinct from meiosis, including mitotic gene conversion (Schmid-Siegert et al., 2017). In perennial, clonally propagated plants, IGH contributes to within-individual genetic variation and influences the long-term stability and uniformity of vegetative propagules, underscoring the importance of understanding its formation (Schoen and Schultz, 2019; Wang et al., 2019; Pineda-Krch and Lehtilä, 2004; Reusch et al., 2021).

Although reports of somatic mutations in plants have increased, mechanistic explanations for stable IGH differences among materials remain limited (Schoen and Schultz, 2019; Schmitt et al., 2022). The accumulation of somatic mutations relies mainly on a balance between mutation generation and repair, with DNA damage response (DDR) and repair systems acting as key determinants (Nisa et al., 2019; Szurman-Zubrzycka et al., 2023). Plants are continuously exposed to DNA-damaging factors, including light, temperature fluctuations, drought and salinity stress, and reactive oxygen species (ROS) (Szurman-Zubrzycka et al., 2023). Repair pathways, such as base excision repair (BER) and homologous recombination (HR), jointly influence whether lesions are repaired efficiently and whether mutations are fixed (Roldán-Arjona and Ariza, 2019; Sakamoto et al., 2021; Grin et al., 2023; Szurman-Zubrzycka et al., 2023). Consequently, the divergence in repair pathway expression and functional differentiation of key genes may contribute to IGH differentiation among materials (Schoen and Schultz, 2019; Wang et al., 2019). However, studies systematically linking DNA-level mutation evidence to coordinated repair pathway changes remain scarce.

*Leymus chinensis* is an important perennial forage grass in arid and semi-arid regions, where it plays a key role in grassland ecosystems and exhibits strong stress tolerance and forage value (Li et al., 2014). Its rhizomatous clonal propagation and prolonged vegetative growth make it suitable for investigating leaf-scale IGH and material stability (Bai et al., 2010). Previous studies have revealed nuclear gene sequence heterogeneity in *L*. *chinensis* leaves and pronounced leaf transcriptomic responses to saline–alkaline stress, indicating both somatic mutation accumulation (Yu et al., 2023b) and high environmental sensitivity (Jin et al., 2008). However, whether leaf variation differs stably among materials with distinct genetic backgrounds and whether such differences are associated with divergence in DNA repair pathways and variation in key repair genes remains insufficiently explored.

Under strictly unified growth conditions, wild *L*. *chinensis* (LC-W) was compared with the cultivated cultivar Zhongke No. 2 (LC-ZK2) and integrated leaf-level evidence from multi-locus amplicon-scale DNA variation, *de novo* transcriptome-derived DDR/BER/HR pathway signatures, and domain-level candidate variation in the BER scaffold gene XRCC1. XRCC1 organized the BER and single-strand break repair and was implicated in active DNA demethylation in plants (Martínez-Macías et al., 2013; Li et al., 2015a). Given the role of BRCT domains as interaction and recruitment modules in DDR proteins (Rodriguez and Songyang, 2008; Leung and Glover, 2011), variations within the XRCC1 BRCT region represent a plausible source of candidate sites. The main research objectives of this study were to: (1) validate the differences in leaf mutation patterns between LC-W and LC-ZK2, (2) characterize the transcriptomic divergence in DDR and repair pathways while identifying key hub genes, and (3) provide candidate mutation sites in the XRCC1 BRCT domain with testable mechanistic hypotheses. Overall, the findings establish an evidence chain linking DNA-level phenotypes to mechanistic clues for understanding leaf IGH divergence in *L*. *chinensis* and provide a basis for subsequent functional validation and stability-oriented breeding evaluation.

## 2 Materials and methods

### 2.1 Plant materials and sampling

Mature seeds of *L. chinensis* were collected from the Huhetala grassland, Inner Mongolia, China (111°37′E, 40°50′N; 1040 m a.s.l.) in late July 2020. Seeds of the cultivated cultivar Zhongke No. 2 were purchased in April 2020 from KeTa Grass Industry Co., Ltd. (Chifeng, Inner Mongolia, China). They were surface sterilized in 30% sodium hypochlorite (NaClO) for 15 min with constant agitation, rinsed five times with sterile distilled water, vernalized at 4 °C for 3 d, and sown on 0.7% Murashige and Skoog (MS) agar medium supplemented with 1% (w/v) sucrose. Plants were grown under a 12 h light/12 h dark photoperiod (approximately 70 μmol m^−2^ s^−1^) at 22 °C. After 2 weeks on MS medium, seedlings were transferred to soil for an additional 2 weeks, after which the fully expanded leaves were harvested for genomic DNA and RNA extraction.

### 2.2 Genomic DNA extraction and gene cloning

Genomic DNA was extracted from leaf or seed tissues using PlantZol (TransGen Biotech, China), purified by phenol–chloroform extraction and isopropanol precipitation, washed with 70% ethanol, dissolved in water, and stored at −20 °C. For each material, three independent plants were sampled as biological replicates. These plants represented independent individuals rather than clonal units derived from a single mother plant. Owing to the absence of a reference genome for *L. chinensis*, primers were designed using *Aegilops tauschii* homologs (Ensembl Plants) (**Supplementary Table 4**). Target fragments were amplified using KOD-plus polymerase (Toyobo, Japan), gel-verified, cloned into pEASY^®^-Blunt Zero (TransGen Biotech, China), and confirmed by colony PCR. Inserts were sequenced by Sanger sequencing (Sangon Biotech, China), with more than 45 plasmids sequenced per gene fragment and at least 15 independent clones analyzed per gene for each plant. To reduce sporadic PCR or cloning artifacts, the variation was quantified per plant using consistent criteria (Section 2.9), and the conclusions required concordant trends across all three loci.

### 2.3 RNA extraction and cDNA synthesis

Total RNA was isolated from the leaves using TRIzol (Invitrogen, Shanghai, China) and treated with RNase-free DNase I (Vazyme, Nanjing, China). RNA concentration and purity were measured using a NanoDrop 2000, and integrity was assessed using an Agilent 5300 system (RQN). Only RNA samples meeting the thresholds of ≥1 μg total RNA, ≥30 ng μL^−1^, RQN > 6.5, and OD260/280 = 1.8–2.2 were used. First-strand cDNA was synthesized from 1 μg RNA using the RevertAid First Strand cDNA Synthesis Kit (Thermo Scientific, USA), and 2 μL of cDNA was used as the PCR template in a 50 μL reaction.

### 2.4 Library preparation and sequencing

RNA-seq libraries were prepared using the Illumina^®^ Stranded mRNA Prep Ligation Kit with 1 μg of total RNA per sample. Messenger RNA (mRNA) was enriched using oligo(dT) magnetic beads and fragmented using fragmentation buffer. Fragmented RNA was reverse-transcribed with random hexamer primers to generate double-stranded cDNA, followed by end repair, phosphorylation, and adaptor ligation, according to the library preparation protocol. The libraries were size-selected with magnetic beads for 300–400 bp inserts and PCR-amplified for 10–15 cycles. After quantification with Qubit 4.0, libraries were sequenced on the NovaSeq X Plus platform using a NovaSeq Reagent Kit to generate paired-end 150 bp reads.

### 2.5 Quality control and *de novo* assembly

Raw paired-end reads were trimmed and quality-controlled using fastp (https://github.com/OpenGene/fastp) with default settings. Clean reads were adopted for *de novo* assembly using Trinity (https://github.com/trinityrnaseq/trinityrnaseq/wiki), and the assembled sequences were filtered with CD-HIT (http://weizhongli-lab.org/cd-hit/) and TransRate (http://hibberdlab.com/transrate/) to enhance assembly quality. Assembly completeness was assessed using BUSCO (Benchmarking Universal Single-Copy Orthologs; http://busco.ezlab.org). Given that *Leymus chinensis* is an outcrossing allotetraploid species, short-read *de novo* assembly may not fully resolve closely related homoeologous, haplotypic or allelic transcripts. Therefore, in this study, transcriptome data were interpreted mainly at the levels of overall differential expression, pathway enrichment, and candidate gene identification. A *de novo* assembly strategy was adopted as a practical approach to retain transcript information from the study materials themselves.

### 2.6 Differential expression analysis and functional analysis

The assembled transcripts were annotated by alignment against public databases, including the NCBI non-redundant protein database (NR), Swiss-Prot, Pfam, eggNOG, Gene Ontology (GO), and the Kyoto Encyclopedia of Genes and Genomes (KEGG) (Kanehisa and Goto, 2000). For expression analysis, clean reads were mapped back to the assembled transcript set using Bowtie2 (Langmead and Salzberg, 2012), and transcript abundance was estimated with RSEM (Li and Dewey, 2011) (http://deweylab.github.io/RSEM/). Gene expression levels were summarized as read counts for downstream differential expression analysis. Differential expression analysis was performed using DESeq2 (Love et al., 2014) (http://bioconductor.org/packages/stats/bioc/DESeq2/), with log_2_ fold change (log_2_FC) defined as log_2_(LC-W/LC-ZK2); genes with |log_2_FC| ≥ 1 and false discovery rate (FDR) < 0.05 were considered differentially expressed genes (DEGs). GO and KEGG enrichment analyses were performed to identify DEGs significantly overrepresented relative to the whole-transcriptome background, using a Bonferroni-corrected P value < 0.05. GO and KEGG enrichment analyses were carried out using GOATOOLS (Klopfenstein et al., 2018) (https://github.com/tanghaibao/GOatools) and Python scipy (Virtanen et al., 2020) (https://scipy.org/), respectively.

### 2.7 qRT-PCR validation

To validate the directionality of RNA-seq-inferred expression changes, qRT-PCR was performed to quantify the relative expression levels of the core BER pathway genes. RNA extraction, reverse transcription, and qPCR amplification were performed following the manufacturer’s protocols, with the primer information presented in **Supplementary Table 4**. Relative expression was calculated using the 2^−ΔΔCt^ method. Because qRT-PCR was performed using independently collected samples with a replicate design that did not fully match RNA-seq, the results were used to assess the expression direction consistency rather than to reproduce RNA-seq effect sizes.

### 2.8 Histochemical detection of ROS

Leaves at the same developmental stage and with similar physiological status were collected from LC-W and LC-ZK2, with three biological replicates for each material. For DAB staining, leaves were vacuum-infiltrated with 1 mg/mL DAB solution (Coolaber, Beijing, China) incubated at 28 °C in the dark for at least 12 h, and then decolorized in 80% ethanol in boiling water until chlorophyll was removed. For NBT staining, leaves were vacuum-infiltrated with 1 mg/mL NBT solution (Coolaber, Beijing, China), incubated at 37 °C overnight, They were then decolorized in 95% ethanol at 80 °C, with the ethanol replaced every 10 min until the green color disappeared. The stained leaves were photographed after destaining. DAB staining was used to detect H_2_O_2_, and NBT staining was used to detect O_2_^-^ accumulation.

### 2.9 Targeted amplification, cloning, and sanger sequencing of the XRCC1 BRCT domain

To profile the variation in the XRCC1 BRCT domain, three plants from each material (LC-W and LC-ZK2) were analyzed. Leaf RNA was extracted and reverse-transcribed to cDNA. A BRCT-containing fragment (CDS 171–411; 240 bp) was amplified using gene-specific primers based on the XRCC1 unigene (F: GATGGGGTGGTCTTTGTGCTG; R: CCAGCATGCATAAGGTAAGGCTC) with high-fidelity KOD-plus polymerase (Toyobo, Japan). The PCR products were gel-verified, cloned into pEASY^®^-Blunt Zero (TransGen Biotech, China), screened by colony PCR, and sequenced using Sanger sequencing (Sangon Biotech, China). At least 15 independent clones were sequenced per individual.

### 2.10 Sequence analysis and BRCT domain characterization

Chromatograms were manually inspected, and questionable sites were re-checked. DNA and translated protein sequences were aligned using ClustalW v1.2.1 and visualized using Jalview v2.11.5. Identical clones were merged as haplotypes, and frequencies were counted per individual. Polymorphic sites, nucleotide diversity (π), and haplotype diversity (Hd) with the haplotype number were calculated in DnaSP v6.12.03 (default settings), treating consecutive deletions at the same position as a single event.

For XRCC1 BRCT, conservation and secondary structure annotations were generated using ESPript 3.2 (accessed 2025-12-06). An NJ tree was built in MEGA v12.1 and visualized in iTOL (accessed 2025-12-06). BRCT haplotype sequences (XRCC1 59–137 aa) were modeled using AlphaFold (accessed 2025-12-30), and structures were superposed and mapped in ChimeraX v1.11.

## 3 Results

### 3.1 LC-ZK2 exhibits a lower mutation burden than LC-W across three nuclear genes in leaves

To compare leaf-scale sequence variation between LC-W and LC-ZK2, three independent plants per material were analyzed as biological replicates using PCR amplification, cloning, and Sanger sequencing of three nuclear genes (**Supplementary Figure 1**; 15 clones per plant). These loci were randomly selected from candidate nuclear genes that showed stable amplification, clear single PCR bands, and high sequencing quality. We were used as representative loci for multi-locus comparison between the two materials. Polymorphic sites (S) and pairwise nucleotide diversity (π) were calculated for each individual. Across all three loci, LC-ZK2 consistently exhibited lower S and π values than LC-W, indicating lower leaf-scale sequence heterogeneity in the cultivated material at the leaf amplicon level.

**Figure 1:**
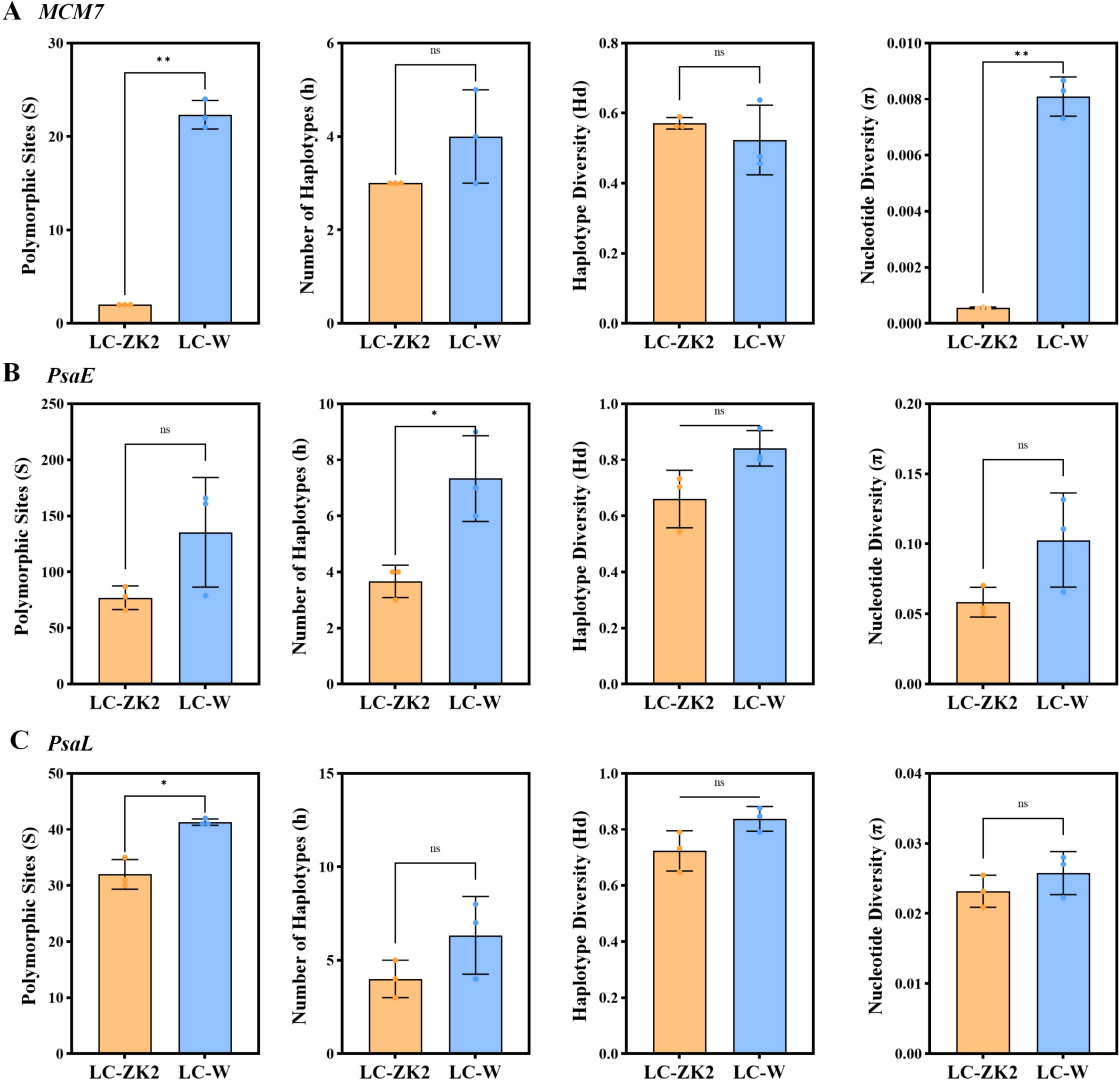
Amplicon cloning–Sanger sequencing reveals lower leaf-level variation in LC-ZK2 than in LC-W across three nuclear loci. **(A–C)** Clone-based amplicon statistics for three nuclear genes (MCM7, PsaE, and PsaL) from leaf samples, including polymorphic sites (S), haplotypes (h), haplotype diversity (Hd), and nucleotide diversity (π). Bars represent mean values across three biological replicates for each material (LC-ZK2 and LC-W), with error bars indicating the variation among replicates. LC-ZK2 consistently exhibits lower S and π values than LC-W. Statistical significance between LC-ZK2 and LC-W was evaluated using a two-tailed Student’s t-test. ns, not significant; *P < 0.05; **P < 0.01.

For PsaL, S in LC-ZK2 (32.0 ± 2.65) was lower than that in LC-W (41.33 ± 0.58), with a corresponding reduction in nucleotide diversity (π; LC-ZK2: 0.02320 ± 0.00228; LC-W: 0.02578 ± 0.00308) (**Figure 1A**). A larger difference was observed for PsaE, for which LC-ZK2 showed S = 77.0 ± 10.54 compared with 135.33 ± 48.85 in LC-W, accompanied by a parallel decrease in π (LC-ZK2: 0.05846 ± 0.01058; LC-W: 0.10278 ± 0.03360) (**Figure 1B**). For MCM7, S in LC-ZK2 was nearly fixed at a low level (2.00), far below that in LC-W (22.33 ± 1.53), and π was similarly reduced (LC-ZK2: 0.00056 ± 0.00003; LC-W: 0.00810 ± 0.00070) (**Figure 1C**). Haplotype-based metrics (h and Hd) showed consistent or weaker locus-specific differences. However, they did not alter the overall inference that all three genes consistently supported the lower leaf-scale sequence heterogeneity observed in LC-ZK2 based on S and π.

We summarized the polymorphic sites detected at each locus into shared and plant-specific categories under a unified alignment framework (**Table 1**). Across the three loci, LC-W generally showed higher total numbers of polymorphic sites than LC-ZK2, particularly for PsaE (167 vs 66) and MCM7 (26 vs 4), whereas the difference for PsaL was smaller (56 vs 48). Shared sites across all three plants or across two plants were observed in both materials, and plant-specific sites were also present. Under the current data framework, these categories are presented as leaf-scale heterogeneous sites rather than as fully confirmed somatic mutations, but they nevertheless provide a clearer material-level summary of the overall difference in sequence heterogeneity between LC-W and LC-ZK2.

**Table 1:** Summary of leaf-scale heterogeneous sites at three nuclear loci in LC-W and LC-ZK2.

Gene	Material	Common callable sites among 3 plants	Total polymorphic sites across 3 plants	Shared by all 3 plants	Shared by 2 plants only	Plant-specific sites (Total)
MCM7	LC-ZK2	1129	4	1	0	3
	LC-W	1129	26	20	1	5
PsaE	LC-ZK2	646	66	35	27	4
	LC-W	647	167	53	83	31
PsaL	LC-ZK2	717	48	15	13	20
	LC-W	717	56	24	14	18

A site was counted as polymorphic within a plant when at least two valid nucleotides were observed among the 15 cloned sequences from that plant. Shared/site-specific classifications were performed only on positions with valid nucleotide calls in all three compared plants within each material. Therefore, the number of common comparable sites may differ slightly between materials because positions containing gaps or missing bases in any one of the three compared plants were excluded from within-material shared/site-specific classification. Because the current data do not allow all variable sites to be rigorously assigned to confirmed somatic mutations, these sites are described here as leaf-scale heterogeneous sites under a unified alignment framework.

Overall, clone-based amplicon sequencing across three nuclear genes and three biological replicates per material showed consistently lower polymorphic site numbers and nucleotide diversity in LC-ZK2 than in LC-W. These results support lower leaf-scale sequence heterogeneity in LC-ZK2 and provide a foundation for exploring the molecular correlates of this phenotype.

### 3.2 Synopsis of RNA-seq analysis

**Figure 2:**
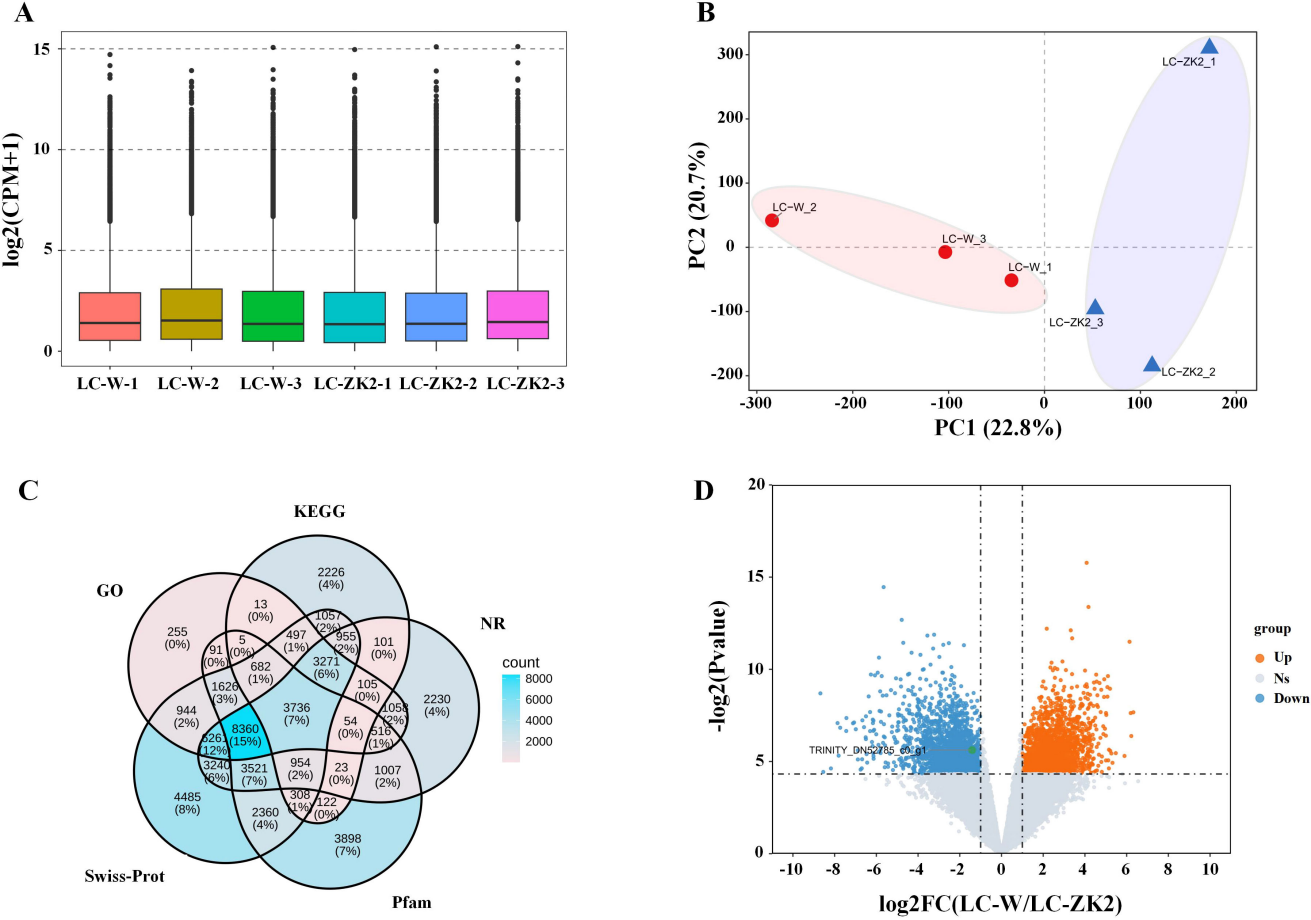
Transcriptome quality assessment, functional annotation overview, and differential expression between LC-W and LC-ZK2. **(A)** Boxplot of expression distributions across samples [log2(CPM + 1)], showing the overall consistency among three biological replicates of LC-W and LC-ZK2. **(B)** Principal component analysis (PCA) based on a global gene expression matrix. Each point represents an individual sample, and ellipses indicate within-group clustering ranges for visualization. **(C)** Venn diagram showing overlaps among unigene annotations in NR, Swiss-Prot, Pfam, GO, and KEGG. **(D)** Volcano plot showing log2FC(LC-W/LC-ZK2) on the x-axis and −log2P-value on the y-axis. Dashed lines denote significance thresholds, and colors indicate significantly upregulated, downregulated, and non-significant genes.Abbreviations: CPM, counts per million; GO, Gene Ontology; KEGG, Kyoto Encyclopedia of Genes and Genomes; NR, NCBI non-redundant; Pfam, protein families.

To characterize molecular signals associated with the DNA-level variation differences described above, RNA-seq analysis was performed on leaves of LC-W and LC-ZK2, with three biological replicates per material. Each library generated approximately 46–54 million raw reads, and 46–53 million clean reads were retained after quality control (**Supplementary Table 1**). The sequencing quality was stable across all samples, with consistently high Q20/Q30 values (**Supplementary Table 1**). The boxplots indicate comparable global expression levels among samples (**Figure 2A**), and PCA clearly separated LC-W from LC-ZK2 with tight within-group clustering (**Figure 2B**), indicating excellent replicate consistency and a strong between-group signal.

Because a reference genome was unavailable, clean reads from all samples were pooled for *de novo* assembly. Trinity generated 280,166 transcripts and 158,997 unigenes (**Supplementary Table 2**). The assembly continuity and completeness metrics (N50/E90N50 and BUSCO) supported the suitability of this transcriptome for functional annotation and differential expression analyses (**Supplementary Table 2**). The annotation coverage across multiple databases (NR, Swiss-Prot, Pfam, GO, and KEGG) was substantial (**Figure 2C**), providing a foundation for enrichment analysis and pathway-level interpretation.

### 3.3 Differentially expressed genes and repair-related enrichment

Differential expression analysis using DESeq2 (|log_2_FC| ≥ 1, FDR < 0.05; log_2_FC = log_2_(LC-W/LC-ZK2)) identified 3,833 DEGs between LC-W and LC-ZK2 leaves. Notably, 1,863 genes were upregulated, and 1,970 genes were downregulated in LC-W relative to LC-ZK2 (**Figure 2D**), indicating a substantial divergence in leaf transcriptional programs.

**Figure 3:**
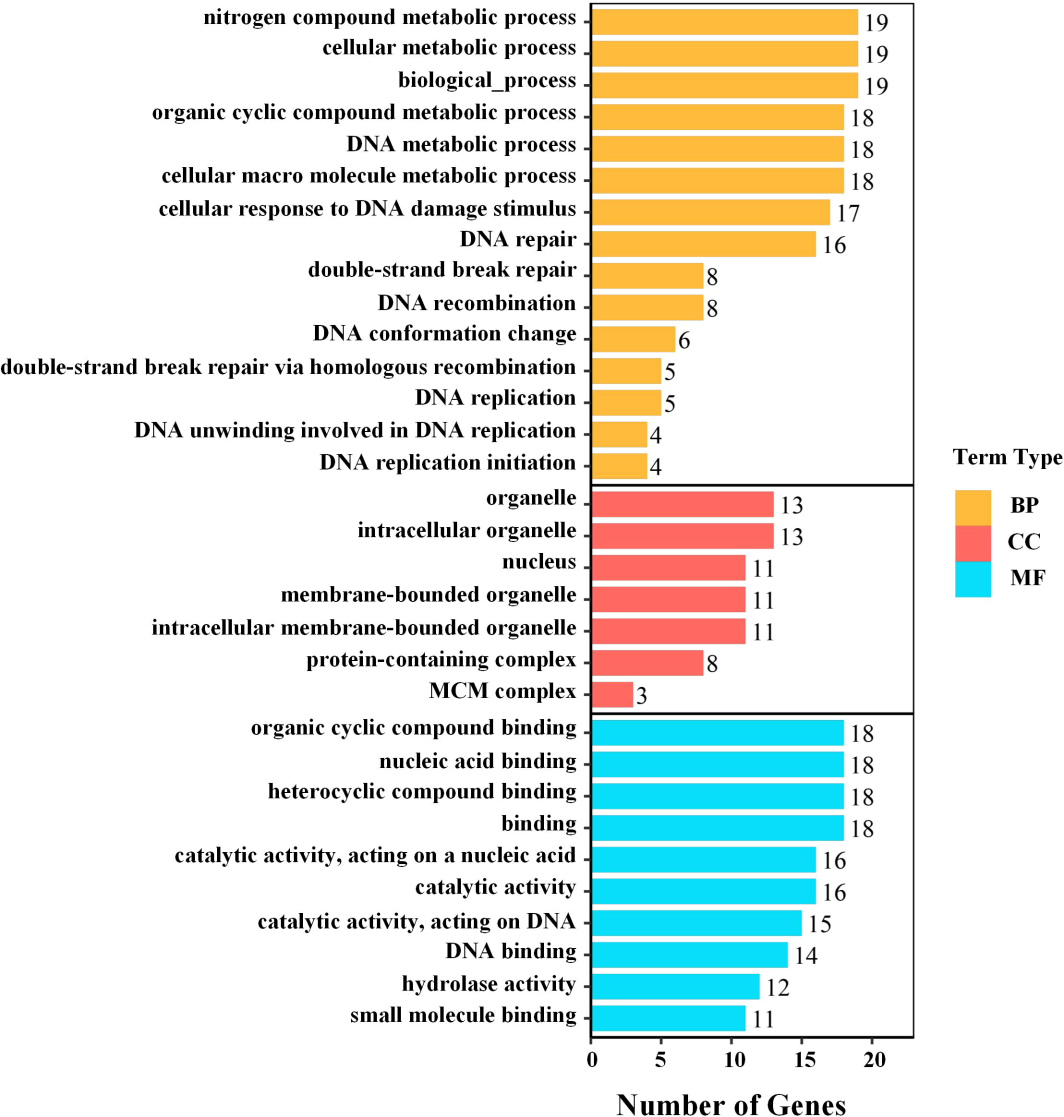
GO enrichment analysis of differentially expressed genes (DEGs). Significantly enriched GO terms are grouped into Biological Process (BP), Cellular Component (CC), and Molecular Function (MF). The bar length indicates the number of DEGs assigned to each term, and the numbers at the end of the bars denote the gene counts. Enrichment significance was corrected for multiple tests (FDR; see Methods). DEGs were identified using DESeq2 (|log2FC| ≥ 1 and FDR < 0.05). The corresponding corrected P values for the GO terms shown here are provided in **Supplementary Table 5**.

**Figure 4:**
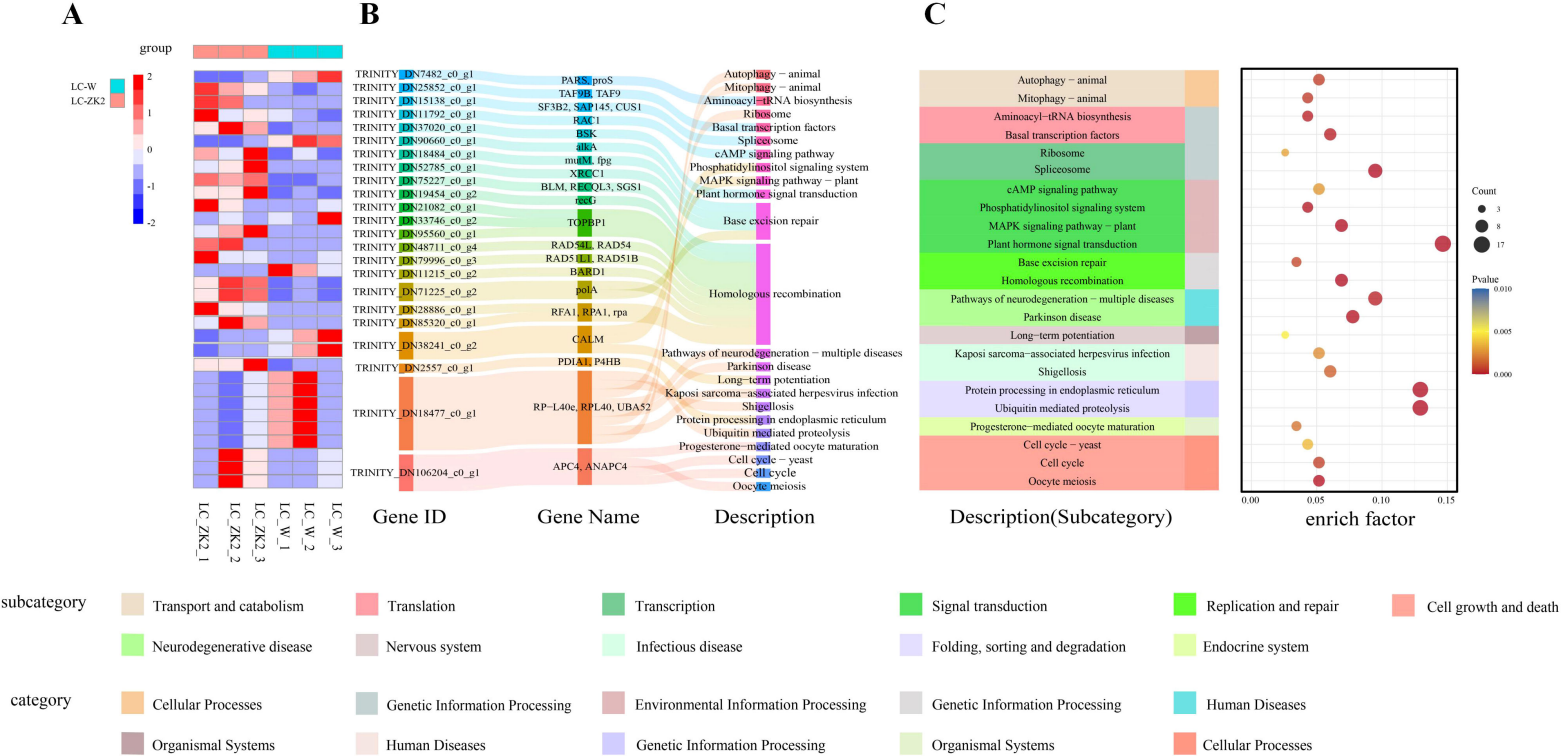
Expression patterns of representative DNA repair-related DEGs and integrated visualization of enriched KEGG pathways. **(A)** Heatmap of representative DEGs involved in base excision repair (BER) and homologous recombination (HR) across biological replicates of LC-W and LC-ZK2. Gene expression values were normalized and row-scaled (Z-scores) for visualization. The colors indicate relatively higher (red) or lower (blue) expression levels. **(B)** Sankey diagram illustrating the associations between representative DEGs and annotated functional modules/pathways. Flow width reflects the relative number of genes contributing to each module. **(C)** KEGG pathway annotation and bubble plot of significantly enriched pathways. The dot size indicates the gene count (Count), and the dot color indicates the adjusted significance (FDR/adjusted p value). The x-axis represents the enrichment factor. DEGs were identified using DESeq2 [|log2FC| ≥ 1, FDR < 0.05; log2FC = log2(LC-W/LC-ZK2)].

GO enrichment analysis indicated that DEGs were significantly overrepresented in genome maintenance–related processes, including DNA repair and cellular responses to DNA damage stimuli, as well as DNA replication–associated terms (**Figure 3**). KEGG analysis similarly highlighted the repair pathways, with particularly strong enrichment of base excision repair (BER) and homologous recombination (HR) (**Figure 4C**), suggesting that the transcriptional differences between LC-W and LC-ZK2 were concentrated in the repair-related pathways.

Consistent with these results, the representative BER- and HR-related genes generally exhibited higher expression in LC-ZK2 (**Figure 4A**), and integrative visualization linked DEGs to coordinated repair-related functional modules (**Figure 4B**). Overall, DEG patterns, functional enrichment, and network-level expression analyses consistently identified DDR and DNA repair program as the major transcriptional features distinguishing LC-W and LC-ZK2 leaves.

### 3.4 XRCC1 as a hub candidate in the BER pathway

**Figure 5:**
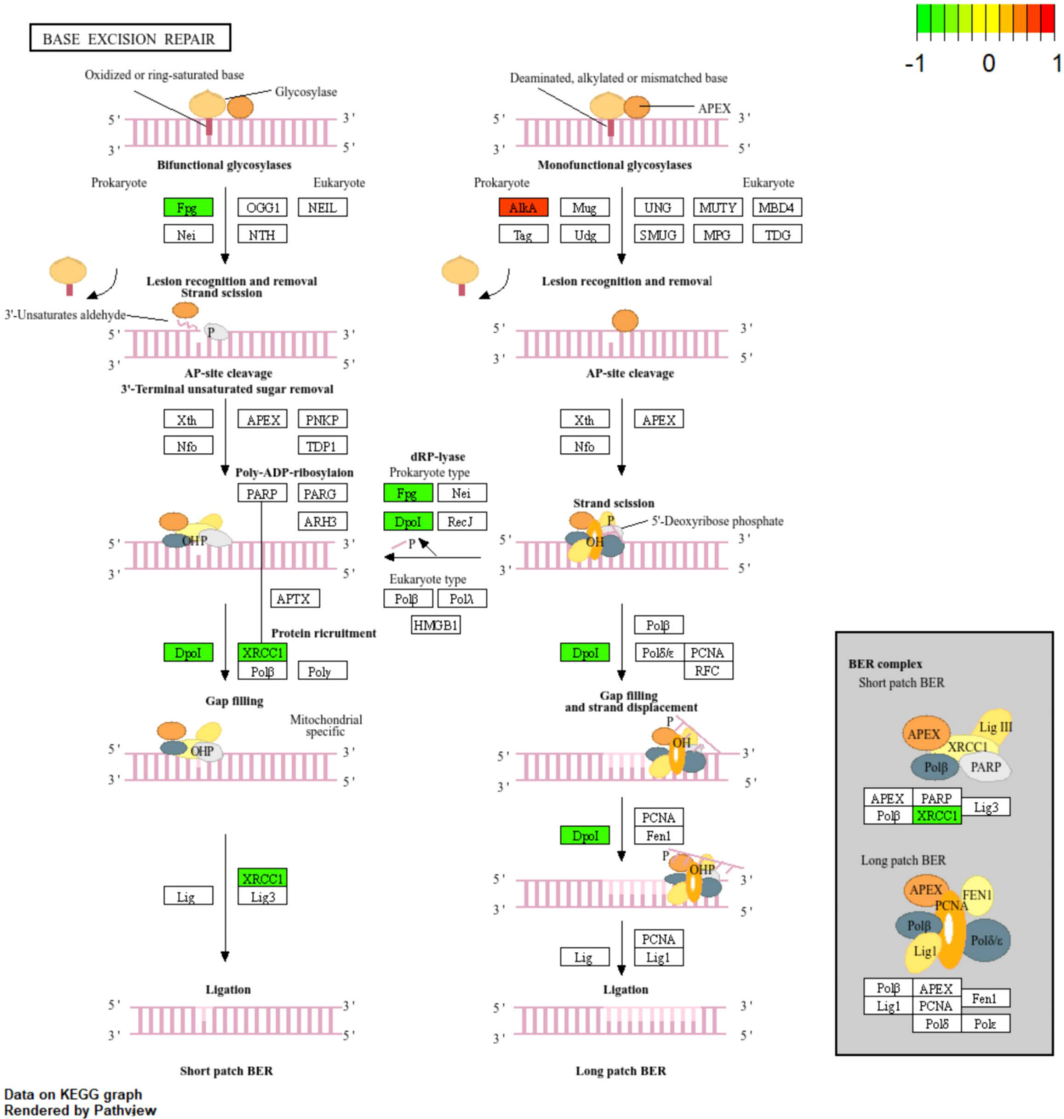
Localization of DEGs in the canonical base excision repair (BER) pathway (KEGG: ko03410).DEGs identified between LC-W and LC-ZK2 were mapped onto the BER pathway and visualized using Pathview. Node colors indicate the direction and magnitude of expression changes (log2FC = log2(LC-W/LC-ZK2)), see color scale): where log2FC>0 indicates upregulation in LC-W, whereas log2FC<0 indicates upregulation in LC-ZK2. Key steps, including lesion recognition/removal, AP-site processing, gap filling, and ligation, are highlighted, along with core components such as XRCC1. DEGs were identified using DESeq2 (|log2FC| ≥ 1 and FDR < 0.05).

To place DEGs within the canonical BER pathway, they were mapped to the KEGG BER pathway (ko03410). Differential signals were distributed across key steps, including lesion recognition and excision, AP-site processing, gap filling, and ligation (**Figure 5**). XRCC1 localized to the gap-processing and ligation stage and exhibited a consistent regulatory direction within the pathway map, supporting its prioritization as a hub candidate (**Figure 5**).

Among repair-related candidate genes, XRCC1 (TRINITY_DN52785_c0_g1) was identified as a prominent candidate hub gene. XRCC1 was significantly upregulated in LC-ZK2, showing an approximately 2.6-fold higher expression than in LC-W (**Figure 2D**), consistent with the overall expression pattern of the BER/HR network (**Figure 4A**). This pattern suggests that XRCC1 may be associated with the observed difference in leaf-scale sequence heterogeneity, consistent with its known role in DNA repair (Martínez-Macías et al., 2013; Roldán-Arjona and Ariza, 2019; Grin et al., 2023).

**Figure 6:**
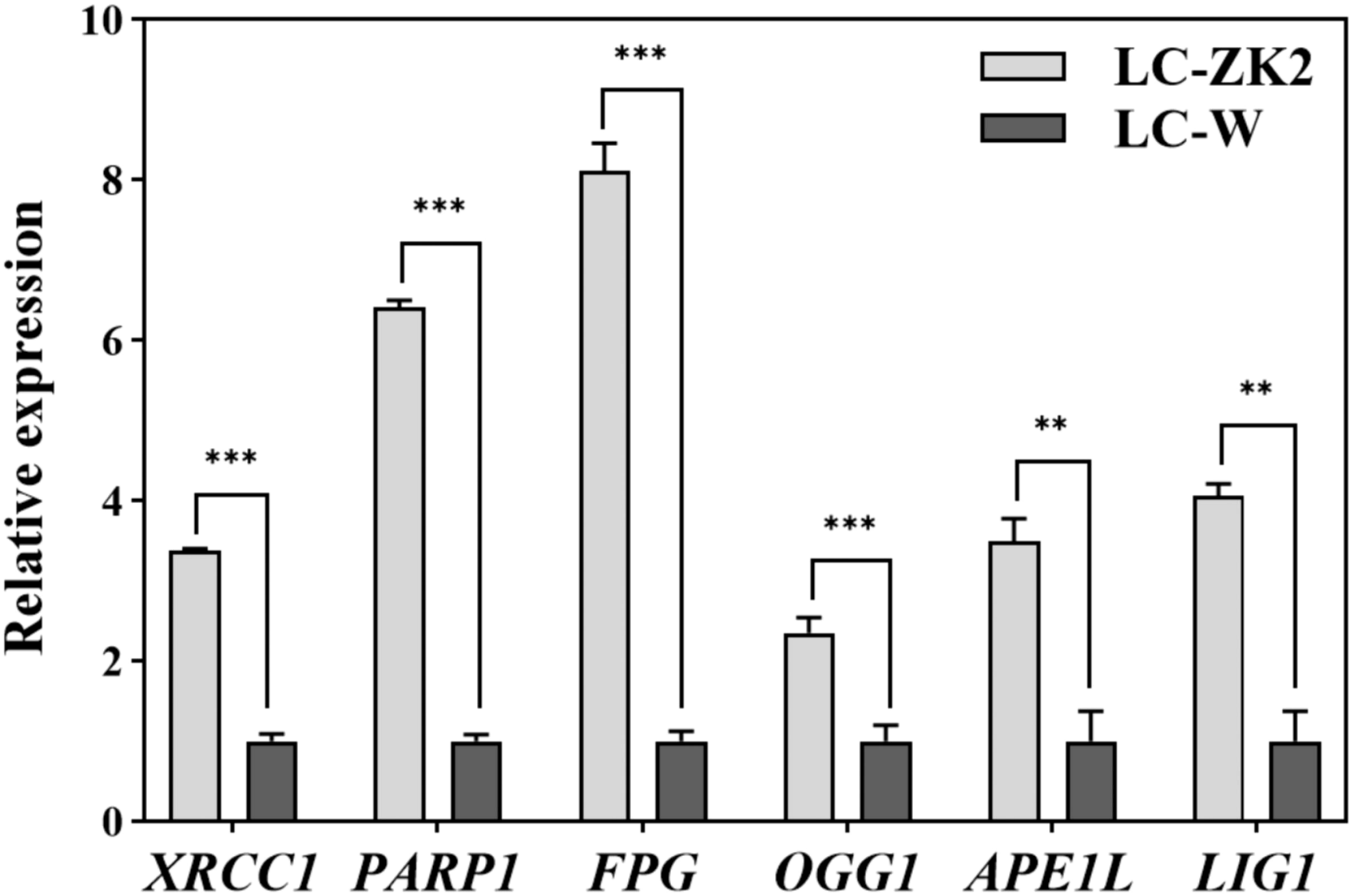
qRT-PCR validation of BER core gene expression in independent samples. Relative expression levels of nine BER core genes (XRCC1, PARP1, FPG, OGG1, APE1L, POLλ, and LIG1) were quantified in leaf samples collected independently from the RNA-seq experiment. Actin was used as the internal reference, and the LC-W group was used as the calibrator (set to 1). Relative expression levels were calculated using the 2−ΔΔCt method. Bars represent mean values, and error bars indicate the standard deviation (SD) of the technical replicates (n = 3). These data were used to assess the directional consistency of gene expression changes with the RNA-seq results. Statistical significance between LC-ZK2 and LC-W was evaluated using a two-tailed Student’s t-test. **P < 0.01; ***P < 0.001.

The directional consistency of RNA-seq trends was evaluated by qRT-PCR analysis of six core BER genes using independently collected leaf samples. Using Actin as the internal reference and LC-W as the calibrator, the qRT-PCR results were consistent with the RNA-seq expression patterns, including for XRCC1 (**Figure 6**), and were intended to confirm the expression direction rather than reproduce RNA-seq effect sizes. Collectively, these transcriptomic results provide mechanistic clues linking DNA repair-related expression patterns, particularly XRCC1-centered BER signals, to the lower leaf-scale sequence heterogeneity observed in LC-ZK2, rather than directly quantifying IGH on a genome-wide scale. To provide additional physiological context, we further performed NBT and DAB staining assays. NBT staining was weaker in LC-ZK2 than in LC-W, whereas DAB staining did not reveal any obvious punctate brown signals in either material (**Supplementary Figure 2**).

### 3.5 cDNA variation and amino-acid substitution sites in the XRCC1 BRCT fragment

**Figure 7:**
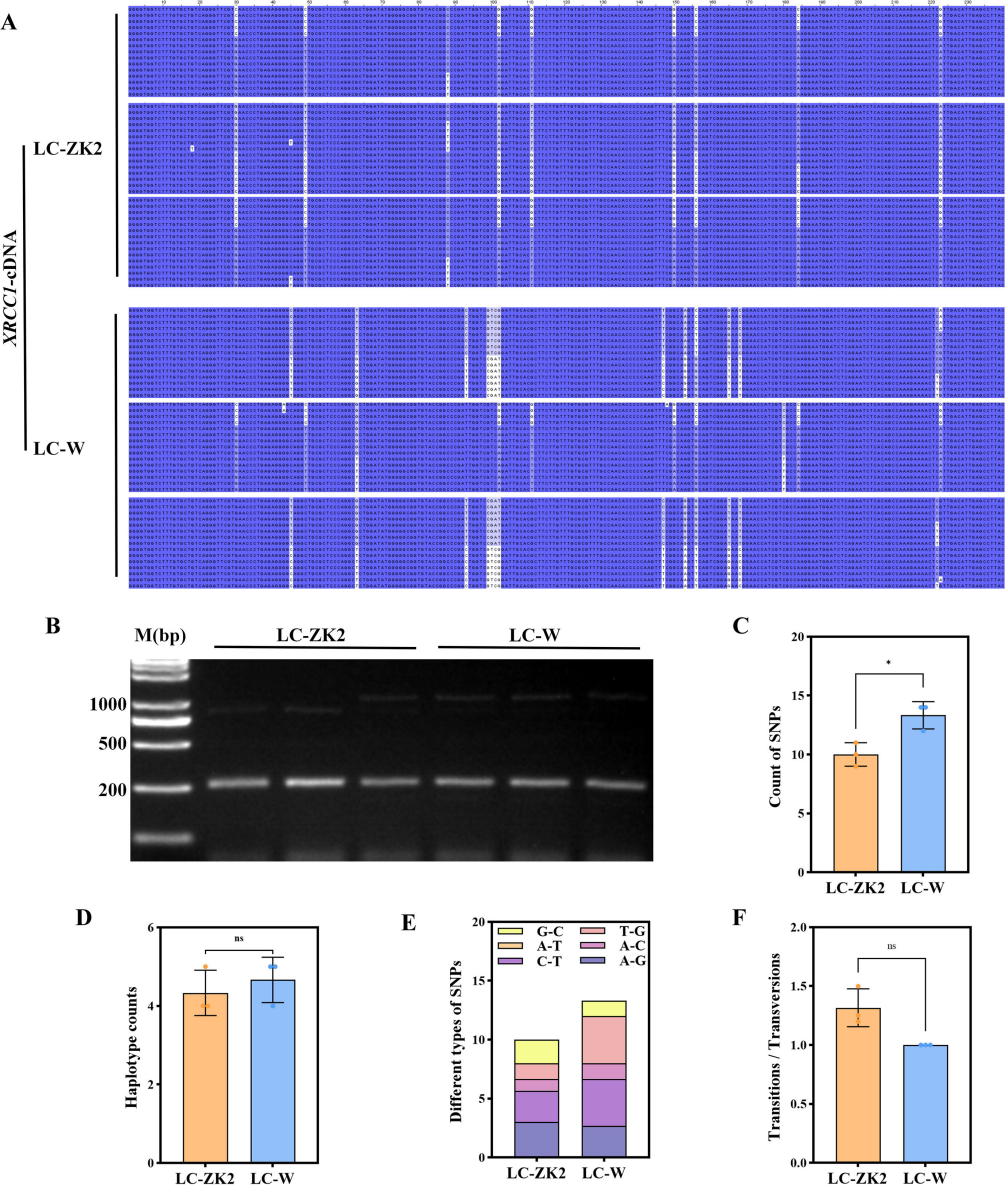
cDNA-level variation in the XRCC1 BRCT fragment differs between LC-ZK2 and LC-W. **(A)** Multiple sequence alignment of clone-derived XRCC1 BRCT cDNA fragments from LC-ZK2 and LC-W. **(B)** Agarose gel electrophoresis showing PCR amplification of the XRCC1 BRCT cDNA fragment. M, DNA ladder. **(C)** Total SNP counts summarized for LC-ZK2 and LC-W. **(D)** Number of haplotypes identified in LC-ZK2 and LC-W. **(E)** Distribution of base substitution types (A↔G, C↔T, A↔C, T↔G, A↔T, and G↔C) in LC-ZK2 and LC-W. **(F)** Transition/transversion (Ts/Tv) ratios summarized for LC-ZK2 and LC-W.

To obtain fragment-level sequence information for XRCC1, the BRCT-containing fragment identified through conserved domain analysis was amplified, cloned, and sequenced using Sanger sequencing. PCR produced bands of the expected size (**Figure 7B**), and sequence alignment revealed multiple variable sites between LC-W and LC-ZK2 within this fragment (**Figure 7A**). Across biological replicates, LC-ZK2 exhibited fewer SNPs (9–11) than LC-W (12–14), whereas the number of haplotypes was comparable between materials (**Figure 7D**). The substitution spectra differed between materials: C→T and T→G substitutions were more frequent in LC-W, whereas the substitution types were more evenly distributed in LC-ZK2 (**Figure 7E**). Consistent with this pattern, the Ts/Tv ratio ranged from 1.20 to 1.50 in LC-ZK2 but was 1.00 for all LC-W plants (**Figure 7F**).

**Figure 8:**
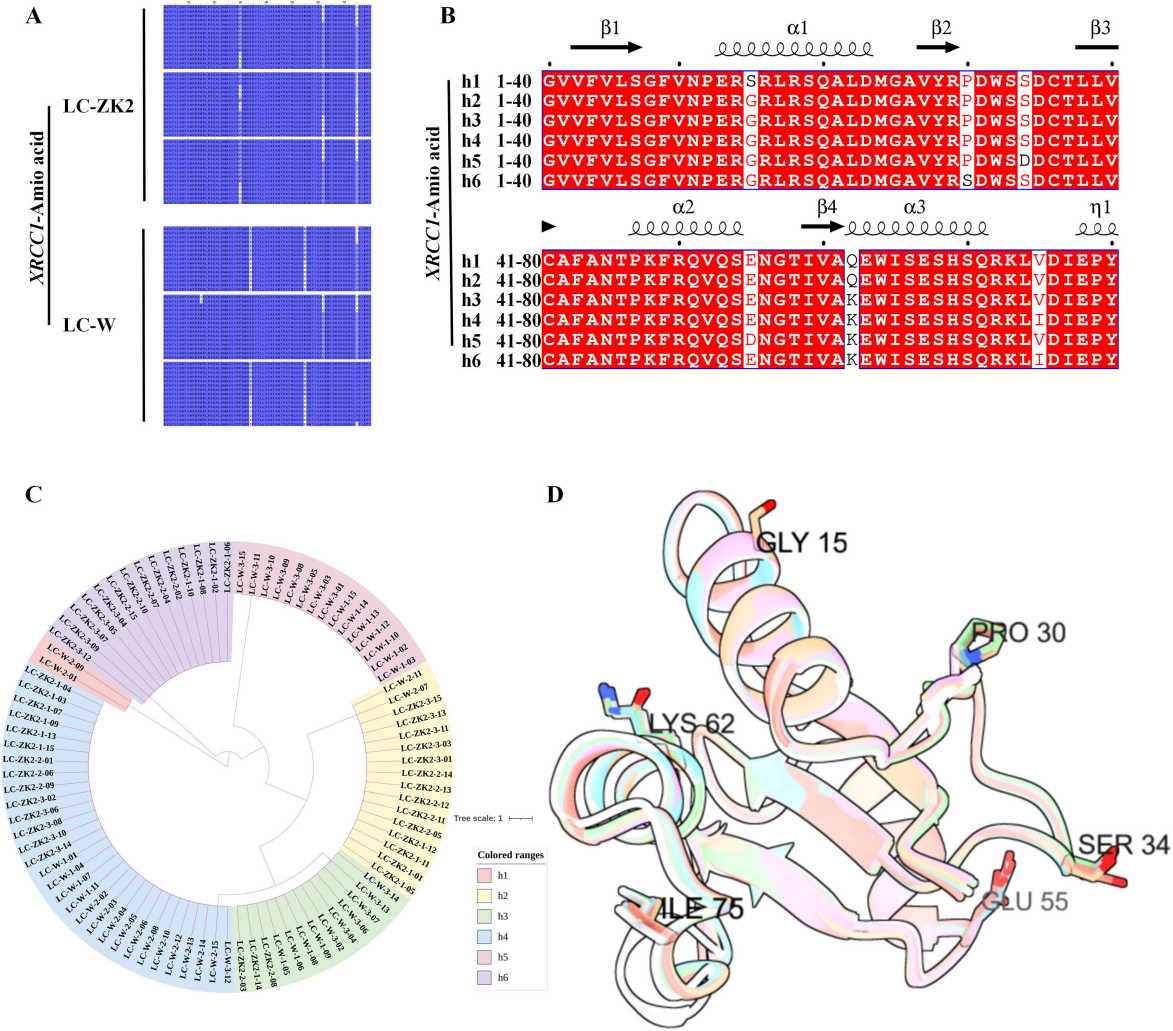
Amino acid-level haplotypes and structural context of XRCC1 BRCT variants. **(A)** Overview of amino acid sequence alignment of XRCC1 BRCT-domain protein haplotypes derived from clone sequences in LC-ZK2 and LC-W. **(B)** Multiple sequence alignment of XRCC1 BRCT domains from representative species, with secondary structural elements (α-helices and β-strands) annotated above the alignment (as indicated). Leymus chinensis haplotype h4 was used as the reference sequence in this study. **(C)** Phylogenetic analysis of BRCT domain amino acid sequences, clustering 60 clone-derived sequences into six protein haplotypes (h1–h6). **(D)** Predicted three-dimensional structures and structural superposition of the BRCT domain for the six protein haplotypes, with variant sites highlighted (positions 15, 30, 34, 55, 62, and 75, based on the numbering scheme used here) to indicate their spatial distribution within the fold. Structural models are used as spatial references for hypothesis generation.

After translation, the sequence variants were mainly concentrated at a limited number of amino acid positions and their substitution combinations (**Figure 8A**), and the nucleotide haplotypes were collapsed into six protein types (h1–h6) (**Figure 8B**; **Supplementary Table 3**). Structural visualization mapped these sites onto the XRCC1 protein context, providing reference points for subsequent validation and experimental design (**Figures 8C, D**; **Supplementary Figure 2**). These results describe the sequence variation patterns within the XRCC1 BRCT fragment and provide candidate sites for downstream analysis. However, they do not by themselves distinguish among somatic variations, inherent genetic background differences, or possible homoeologous sequence contributions.

## 4 Discussion

This study integrated multi-locus, amplicon-based DNA variation with *de novo* transcriptomic signals to propose a candidate mechanistic framework for leaf IGH divergence between *Leymus chinensis* materials under strictly controlled conditions. The conclusions focused on the stable differences detected at the targeted amplicon scale and the associated divergence in repair-related transcriptional programs, rather than on the genome-wide mutation rates or direct measurements of repair efficiency. Accordingly, the findings established an evidence chain linking reproducible DNA-level observations to mechanistic clues. This study defined a stable leaf-level variation phenotype, prioritized candidate repair pathways and hub genes, and identified domain-level candidate sites suitable for subsequent functional validation and causal testing under unified DNA damage conditions.

### 4.1 Multi-locus concordance of leaf variation differences

Across three independent nuclear loci and biological replicates (three plants per material, 15 clones per plant), LC-ZK2 consistently exhibited fewer polymorphic sites (S) and lower nucleotide diversity (π) in leaves than LC-W under strictly controlled conditions (**Figure 1**). Somatic mutations are widespread in plants and can accumulate at low frequencies during growth, as supported by high-accuracy sequencing studies and reviews of trees and *Arabidopsis* (Tomimoto and Satake, 2024; Waneka et al., 2024; Johannes, 2025a). Because plants lack an early and strictly segregated germline (Lanfear, 2018), such mutations are expected to contribute to genetic heterogeneity and generate detectable differences at the organ or branch scales (Schoen and Schultz, 2019; Wang et al., 2019; Reusch et al., 2021). Given the allotetraploid background of *Leymus chinensis*, the present data are more appropriately interpreted as evidence of leaf-scale sequence heterogeneity rather than as a direct genome-wide quantification of IGH or somatic mutation burden. Because *L. chinensis* is an allotetraploid species, the contribution of homoeologous copies to the amplified products cannot be completely excluded. However, the observed patterns are unlikely to be explained solely by fixed homoeologous divergence and are better interpreted as leaf-scale sequence heterogeneity.

The present findings reflect differences in the sequence variation patterns within the targeted amplicons rather than genome-wide mutation patterns. Cellular lineage and tissue development can influence the accumulation and distribution of somatic mutations, such that distinct organs or positions may exhibit different mutation signals (Ji et al., 2025; Scherer et al., 2025). In addition, somatic variant detection is sensitive to analytical thresholds and requires careful interpretation, particularly in complex genetic backgrounds (Schmitt et al., 2022; Xian et al., 2025). If the observed differences can be stably reproduced using larger sample sizes and additional loci, or at the genome scale, they would more convincingly support systematic differences in leaf-scale sequence heterogeneity between the two materials (Tomimoto and Satake, 2023; Schmitt et al., 2024; Johannes, 2025b; Xian et al., 2025). In long-lived perennials, the accumulation and retention of somatic mutations over time further suggest the potential long-term stability and applied relevance of these heterogeneity patterns (Satake et al., 2024). Overall, multi-locus concordant evidence provides a useful starting point for downstream mechanistic exploration.

### 4.2 Pathway-level signals of DNA repair programs

Continuous exposure of leaves to environmental and metabolic sources of DNA damage (e.g., light, temperature fluctuations, and ROS) can cause persistent DNA damage (Nimeth et al., 2020). In this context, the differential expression of DDR and repair pathways between materials represents a plausible explanation for differences in net mutation accumulation (Szurman-Zubrzycka et al., 2023). DDR maintains genomic stability by coordinating cell cycle regulation with multiple DNA repair pathways (Nisa et al., 2019; Kutashev et al., 2024). Chloroplast metabolism is a major source of ROS and can influence nuclear gene expression and stress responses through retrograde signaling (Foyer and Hanke, 2022), whereas chloroplast and mitochondrial genomes require continuous maintenance and repair under oxidative conditions (Mahapatra et al., 2021). This framework is consistent with the view that persistent damage, combined with altered repair activity, increases the likelihood that lesions become fixed as detectable mutations. Consistent with this expectation, our transcriptomic analyses showed significant enrichment of DEGs in DDR- and repair-related processes, with GO/KEGG analyses highlighting the coordinated pathway-level changes, particularly in BER and HR (**Figure 3**).

Concurrently, transcriptional divergence does not necessarily translate into differences in repair efficiency. DDR also regulates cell cycle checkpoints and growth arrest (Takahashi et al., 2019; Herbst et al., 2024; Serrano-Mislata et al., 2025) and is linked to cell fate control in certain cell types (Huerta-Venegas et al., 2024). Accordingly, functional validation under unified damage treatments (e.g., H_2_O_2_, UV, and MMS) is required to assess whether pathway-level expression divergence corresponds to repair capacity (Grin et al., 2023; Szurman-Zubrzycka et al., 2023). Overall, the transcriptomic results identified coordinated divergence in the DDR/BER/HR modules, providing prioritized mechanistic clues and testable hypotheses for the observed amplicon-scale mutation differences. On the other hand, further functional assays under standardized damage conditions are needed to analyze the correspondence with repair capacity.

### 4.3 The hub role of XRCC1

As a scaffold protein in BER and single-strand break repair, XRCC1 coordinates multiple repair factors and influences complex assembly and pathway throughput, which can reflect the repair network-level differences between materials (Martínez-Macías et al., 2013; Roldán-Arjona and Ariza, 2019; Grin et al., 2023). In plants, XRCC1 participates in repair processes associated with active DNA demethylation (Li et al., 2015b, 2015c; Zhang et al., 2022), supporting its broad role in genome maintenance. In this study, XRCC1 was significantly upregulated in LC-ZK2, and the qRT-PCR results confirmed that the expression changes were consistent in the direction with RNA-seq (**Figure 6**). These observations position XRCC1 as a tractable entry point linking pathway-level transcriptional signatures to differences in leaf-scale sequence heterogeneity.

XRCC1 reflects coordinated regulation at the BER network level rather than a single-gene effect. The differences between materials may arise from multi-node regulation and combined repair efficiency (Bourbousse et al., 2018; Yu et al., 2023a; Bao et al., 2025). Accordingly, XRCC1 can be treated as an actionable hub candidate for follow-up functional assays rather than as the sole determinant. Because LC-ZK2 also carries amino acid substitutions in the XRCC1 BRCT-containing fragment, we cannot exclude the possibility that higher XRCC1 expression partly represents a compensatory response rather than direct evidence of enhanced repair efficiency. The weaker NBT staining in LC-ZK2 further suggests that the lower leaf-scale sequence heterogeneity in the cultivated material may be associated with both repair-related expression patterns and a lower superoxide-related oxidative background. In contrast, DAB staining did not reveal a clear difference in H_2_O_2_ level under the present conditions (**Supplementary Figure 2**).

### 4.4 Candidate-site clues in the BRCT region

Using cDNA cloning and sequencing of the XRCC1 BRCT-containing fragment, LC-ZK2 carried fewer SNPs than LC-W within this region, along with differences in substitution spectra and Ts/Tv ratios (**Figure 7**). After translation, multiple nucleotide haplotypes converged into a limited number of protein types, with variation concentrated at a small number of amino acid substitution sites and their combinations. Because BRCT domains function as phosphorylation-dependent interaction and recruitment modules in DDR proteins, such substitutions could potentially influence interaction interfaces and complex assembly (Rodriguez and Songyang, 2008; Leung and Glover, 2011).

However, the BRCT variation evidence in this study was derived from cDNA clone sequencing and did not substitute for confirmation at the genomic DNA level (Verwilt et al., 2023). In addition, structural predictions are not equivalent to functional proofs (Terwilliger et al., 2024). The substantial sharing of variant sites across individuals in the XRCC1 BRCT fragment suggests that the inherent sequence background may contribute to the observed pattern. Therefore, this fragment should be interpreted cautiously as complementary evidence rather than as a standalone indicator of IGH or somatic mutations. Accordingly, the BRCT-region results are presented as candidate-site clues and references for experimental design in subsequent validation rather than as a basis for causal inference.

### 4.5 Limitations and priorities for follow-up validation

This study proposed an association framework linking amplicon-scale DNA variation, pathway-level transcriptomic repair signatures, and XRCC1-BRCT candidate sites. Several limitations remain. First, DNA variation was assessed using three nuclear amplicons, reflecting the fragment-scale mutation burden rather than genome-wide mutation rates. Cloning-based approaches can be sensitive to PCR and amplification artifacts, and future studies should expand loci, apply UMI-enabled amplicon sequencing, or adopt genome-scale strategies. Second, transcriptomic divergence suggests the differentiation of DDR/BER/HR programs but does not directly measure repair efficiency and may be influenced by tissue state and cellular composition. Third, the XRCC1 BRCT variation was inferred from cDNA and may require further confirmation at the gDNA level, and structural prediction alone cannot imply functional impact. Based on these limitations, immediate priorities include validating key BRCT sites at the genomic level and comparing damage sensitivity and repair kinetics between materials under standardized H_2_O_2_, UV, and MMS treatments.

## 5 Conclusions

This study integrated three complementary lines of evidence at the leaf scale in *Leymus chinensis*. First, the analyses of three nuclear gene amplicons across biological replicates consistently supported lower leaf-scale sequence variation metrics (S and π) in LC-ZK2 than in LC-W, supporting a reproducible gene-fragment–level heterogeneity pattern. Second, *de novo* transcriptome profiling revealed coordinated expression divergence in DDR/BER/HR pathways and prioritized XRCC1 as a prominent hub candidate. Third, cDNA clone-based Sanger sequencing of the XRCC1 BRCT-containing fragment generated a condensed haplotype/protein-type map and identified candidate substitution sites. Rather than directly claiming genome-wide IGH differences or a proven causal effect on repair efficiency, these findings were positioned as a testable evidence chain that defined a reproducible phenotype, highlighted prioritized pathways and hub-gene candidates, and provided actionable candidate site resources. Overall, the results lay a foundation for subsequent gDNA validation and functional assays and provide direction for stability-oriented breeding evaluation.

## Data Availability

The data presented in this study are deposited in the NCBI BioProject repository, accession number PRJNA1439292. The corresponding SRA accession numbers are SRR37704692, SRR37704691, SRR37704690, SRR37704689, SRR37704688, and SRR37704687.

## Supplementary material

Supplementary Figure 1 Alignment overview and PCR validation for three nuclear loci used in clone-based variation analysis. **(A)** Multiple sequence alignment snapshots of clone-derived amplicons at three nuclear loci (MCM7, PsaE, and PsaL) in LC-ZK2 and LC-W. Each locus includes three biological replicates per material (labeled 1–3). **(B)** Representative agarose gel images showing successful PCR amplification of MCM7, PsaE, and PsaL in LC-ZK2 and LC-W; M, DNA ladder;

Supplementary Figure 2 NBT and DAB staining of leaves from LC-ZK2 and LC-W. NBT staining was used to detect superoxide anion (O₂⁻), and DAB staining was used to detect hydrogen peroxide (H₂O₂). Representative leaf images are shown for three biological replicates of each material. NBT staining was stronger in LC-W than in LC-ZK2, whereas no obvious punctate brown signals were observed in the DAB-stained leaves of either material under the present conditions. Scale bars = 5 mm.

Supplementary Figure 3 AlphaFold2-predicted structures of the XRCC1 BRCT domain for six amino-acid haplotypes. Predicted BRCT-domain structures for six amino-acid haplotypes (h1–h6) are shown in cartoon representation. Models are colored by per-residue confidence (pLDDT; dark blue, >90; blue, 70–90; light yellow, 50–70; orange, <50). The overall model score (ipTM) is indicated in each panel.

Supplementary Table 1 Summary statistics of RNA-seq data for Leymus chinensis leaf samples.

Supplementary Table 2 Assembly metrics for the de novo transcriptome of Leymus chinensis.

Supplementary Table 3 Effects of XRCC1 BRCT-domain nucleotide substitutions on codons and amino acids.

Supplementary Table 4 Primers used for PCR validation of genes in Leymus chinensis.

Supplementary Table 5 Corrected P values of the GO terms shown in **Figure 3**.

## Glossary

IGH: Intraorganismal genetic heterogeneity
LC-W: wild Leymus chinensis
LC-ZK2: cultivated cultivar Zhongke No. 2
BER: base excision repair
HR: homologous recombination
MCM7: Minichromosome Maintenance Complex Component 7
PsaE: Photosystem I Subunit E
PsaL: Photosystem I Subunit L
XRCC1: X-ray Repair Cross-Complementing Protein 1

## References

[B1] Bai W. Xun F. Li Y. Zhang W. Li L. (2010). Rhizome severing increases root lifespan of Leymus chinensis in a typical steppe of Inner Mongolia. PloS One 5, e12125. doi: 10.1371/journal.pone.0012125. PMID: 20711343 PMC2920826

[B2] Bao W. Deng Z. Zhuang S. Xu C. Yang Z. Zheng M. . (2025). Post-translational modifications of SOG1 enable dynamic control of plant DNA damage response. Sci. Adv. 11, eadw9803. doi: 10.1126/sciadv.adw9803. PMID: 40971436 PMC12448104

[B3] Bourbousse C. Vegesna N. Law J. A. (2018). SOG1 activator and MYB3R repressors regulate a complex DNA damage network in Arabidopsis. Proc. Natl. Acad. Sci. U.S.A. 115, E12453–E12462. doi: 10.1073/pnas.1810582115. PMID: 30541889 PMC6310815

[B4] Foyer C. H. Hanke G. (2022). ROS production and signalling in chloroplasts: cornerstones and evolving concepts. Plant J. 111, 642–661. doi: 10.1111/tpj.15856. PMID: 35665548 PMC9545066

[B5] Goel M. Campoy J. A. Krause K. Baus L. C. Sahu A. Sun H. . (2024). The vast majority of somatic mutations in plants are layer-specific. Genome Biol. 25, 194. doi: 10.1186/s13059-024-03337-0. PMID: 39049052 PMC11267851

[B6] Grin I. R. Petrova D. V. Endutkin A. V. Ma C. Yu B. Li H. . (2023). Base excision DNA repair in plants: Arabidopsis and beyond. Int. J. Mol. Sci. 24, 14746. doi: 10.3390/ijms241914746. PMID: 37834194 PMC10573277

[B7] Herbst J. Li Q. Q. De Veylder L. (2024). Mechanistic insights into DNA damage recognition and checkpoint control in plants. Nat. Plants 10, 539–550. doi: 10.1038/s41477-024-01652-9. PMID: 38503962

[B8] Huerta-Venegas E. Raya-González J. Ruíz-Herrera L. F. López-Bucio J. (2024). Phytochrome A controls the DNA damage response and cell death tolerance within the Arabidopsis root meristem. Plant Cell Environ. 47, 2797–2813. doi: 10.1111/pce.14912. PMID: 38251425

[B9] Ji Y. Chen X. Zhang X. Wang W. Xue L. Zhong Y. . (2025). Mutations mark cell lineages and sectors in flowers of a woody angiosperm. PloS Genet. 21, e1011829. doi: 10.1371/journal.pgen.1011829. PMID: 40825064 PMC12370204

[B10] Jia X. Zhang Q. Jiang M. Huang J. Yu L. Traw M. B. . (2021). Mitotic gene conversion can be as important as meiotic conversion in driving genetic variability in plants and other species without early germline segregation. PloS Biol. 19, e3001164. doi: 10.1371/journal.pbio.3001164. PMID: 33750968 PMC8016264

[B11] Jin H. Kim H. R. Plaha P. Liu S. K. Park J. Y. Piao Y. Z. . (2008). Expression profiling of the genes induced by Na_2_CO_3_ and NaCl stresses in leaves and roots of Leymus chinensis. Plant Sci. 175, 784–792. doi: 10.1016/j.plantsci.2008.07.016. PMID: 41872344

[B12] Johannes F. (2025a). Allometric scaling of somatic mutation and epimutation rates in trees. Evolution 79, 1–5. doi: 10.1093/evolut/qpae150. PMID: 39432579

[B13] Johannes F. (2025b). Somatic evolution of stem cell mutations in long-lived plants. Mol. Biol. Evol. 42, msaf165. doi: 10.1093/molbev/msaf165. PMID: 40614173 PMC12342775

[B14] Kanehisa M. Goto S. (2000). KEGG: kyoto encyclopedia of genes and genomes. Nucleic Acids Res. 28, 27–30. doi: 10.1093/nar/28.1.27. PMID: 10592173 PMC102409

[B15] Klopfenstein D. V. Zhang L. Pedersen B. S. Ramírez F. Warwick Vesztrocy A. Naldi A. . (2018). GOATOOLS: A Python library for Gene Ontology analyses. Sci. Rep. 8, 10872. doi: 10.1038/s41598-018-28948-z. PMID: 30022098 PMC6052049

[B16] Kutashev K. Meschichi A. Reeck S. Fonseca A. Sartori K. White C. I. . (2024). Differences in RAD51 transcriptional response and cell cycle dynamics reveal varying sensitivity to DNA damage among Arabidopsis thaliana root cell types. New Phytol. 243(3):966–980. doi: 10.1111/nph.19875. PMID: 38840557

[B17] Lanfear R. (2018). Do plants have a segregated germline? PloS Biol. 16, e2005439. doi: 10.1371/journal.pbio.2005439. PMID: 29768400 PMC5973621

[B18] Langmead B. Salzberg S. L. (2012). Fast gapped-read alignment with Bowtie 2. Nat. Methods 9, 357–359. doi: 10.1038/nmeth.1923. PMID: 22388286 PMC3322381

[B19] Leung C. C. Y. Glover J. N. M. (2011). BRCT domains: Easy as one, two, three. Cell. Cycle 10, 2461–2470. doi: 10.4161/cc.10.15.16312. PMID: 21734457 PMC3180187

[B20] Li B. Dewey C. N. (2011). RSEM: accurate transcript quantification from RNA-Seq data with or without a reference genome. BMC Bioinf. 12, 323. doi: 10.1186/1471-2105-12-323. PMID: 21816040 PMC3163565

[B21] Li Q. Wang X. Sun H. Zeng J. Cao Z. Li Y. . (2015a). Regulation of active DNA demethylation by a methyl-CpG-binding domain protein in Arabidopsis thaliana. PloS Genet. 11, e1005210. doi: 10.1371/journal.pgen.1005210. PMID: 25933434 PMC4416881

[B22] Li X. Wang J. Lin J. Wang Y. Mu C. (2014). Rhizomes help the forage grass Leymus chinensis to adapt to the salt and alkali stresses. Sci. World J. 2014, 213401. doi: 10.1155/2014/213401. PMID: 25121110 PMC4121218

[B23] Li Y. Córdoba-Cañero D. Qian W. Zhu X. Tang K. Zhang H. . (2015b). An AP endonuclease functions in active DNA demethylation and gene imprinting in Arabidopsis. PloS Genet. 11, e1004905. doi: 10.1371/journal.pgen.1004905. PMID: 25569774 PMC4287435

[B24] Li Y. Duan C. G. Zhu X. Qian W. Zhu J. K. (2015c). A DNA ligase required for active DNA demethylation and genomic imprinting in Arabidopsis. Cell Res. 25, 757–760. doi: 10.1038/cr.2015.45. PMID: 25906993 PMC4456619

[B25] Love M. I. Huber W. Anders S. (2014). Moderated estimation of fold change and dispersion for RNA-seq data with DESeq2. Genome Biol. 15, 550. doi: 10.1186/s13059-014-0550-8. PMID: 25516281 PMC4302049

[B26] Mahapatra K. Banerjee S. De S. Mitra M. Roy P. Roy S. (2021). An insight into the mechanism of plant organelle genome maintenance and implications of organelle genome in crop improvement: an update. Front. Cell Dev. Biol. 9. doi: 10.3389/fcell.2021.671698. PMID: 34447743 PMC8383295

[B27] Martínez-Macías M. I. Córdoba-Cañero D. Ariza R. R. Roldán-Arjona T. (2013). The DNA repair protein XRCC1 functions in the plant DNA demethylation pathway by stimulating cytosine methylation excision, gap tailoring, and DNA ligation. J. Biol. Chem. 288, 5496–5505. doi: 10.1074/jbc.M112.427617. PMID: 23316050 PMC3581409

[B28] Nimeth B. A. Riegler S. Kalyna M. (2020). Alternative splicing and DNA damage response in plants. Front. Plant Sci. 11. doi: 10.3389/fpls.2020.00091. PMID: 32140165 PMC7042379

[B29] Nisa M. U. Huang Y. Benhamed M. Raynaud C. (2019). The plant DNA damage response: Signaling pathways leading to growth inhibition and programmed cell death. Front. Plant Sci. 10. doi: 10.3389/fpls.2019.00653. PMID: 31164899 PMC6534066

[B30] Pineda-Krch M. Lehtilä K. (2004). Costs and benefits of genetic heterogeneity within organisms. J. Evol. Biol. 17, 1167–1177. doi: 10.1111/j.1420-9101.2004.00808.x. PMID: 15525396

[B31] Reusch T. B. H. Baums I. B. Werner B. (2021). Evolution via somatic genetic variation in modular species. Trends Ecol. Evol. 36, 1083–1092. doi: 10.1016/j.tree.2021.08.011. PMID: 34538501

[B32] Rodriguez M. C. Songyang Z. (2008). BRCT domains: Phosphopeptide binding and signaling modules. Front. Biosci. 13, 5905–5915. doi: 10.2741/3125. PMID: 18508631

[B33] Roldán-Arjona T. Ariza R. R. (2019). Base excision repair in plants: An unfolding story with familiar and novel characters. Front. Plant Sci. 10. doi: 10.3389/fpls.2019.01055. PMID: 31543887 PMC6728418

[B34] Sakamoto A. N. Sakamoto T. Yokota Y. Teranishi M. Yoshiyama K. O. Kimura S. (2021). SOG1, a plant-specific master regulator of DNA damage responses, originated from nonvascular land plants. Plant Direct 5, e370. doi: 10.1002/pld3.370. PMID: 34988354 PMC8711748

[B35] Satake A. Imai R. Fujino T. Tomimoto S. Ohta K. Na’iem M. . (2024). Somatic mutation rates scale with time not growth rate in long-lived tropical trees. eLife 12, RP88456. doi: 10.7554/eLife.88456. PMID: 39441734 PMC11498935

[B36] Scherer J. Hinczewski M. Nelms B. (2025). Quantitative and sensitive sequencing of somatic mutations in plants. Proc. Natl. Acad. Sci. U.S.A. 122, e2426650122. doi: 10.1073/pnas.2426650122. PMID: 40768352 PMC12358873

[B37] Schmid-Siegert E. Sarkar N. Iseli C. Calderon S. Gouhier-Darimont C. Chrast J. . (2017). Low number of fixed somatic mutations in a long-lived oak tree. Nat. Plants 3, 926–929. doi: 10.1038/s41477-017-0066-9. PMID: 29209081

[B38] Schmitt S. Heuret P. Troispoux V. Beraud M. Cazal J. Chancerel É. . (2024). Low-frequency somatic mutations are heritable in tropical trees Dicorynia guianensis and Sextonia rubra. Proc. Natl. Acad. Sci. U.S.A. 121, e2313312121. doi: 10.1073/pnas.2313312121. PMID: 38412128 PMC10927512

[B39] Schmitt S. Leroy T. Heuertz M. Tysklind N. (2022). Somatic mutation detection: A critical evaluation through simulations and reanalyses in oaks. Peer Community J. 2, e68. doi: 10.24072/pcjournal.187. PMID: 41369854

[B40] Schoen D. J. Schultz S. T. (2019). Somatic mutation and evolution in plants. Annu. Rev. Ecol. Evol. Syst. 50, 49–73. doi: 10.1146/annurev-ecolsys-110218-024955. PMID: 41139587

[B41] Serrano-Mislata A. Hernández-García J. de Ollas C. Blanco-Touriñán N. Jurado-García S. Úrbez C. . (2025). Growth arrest is a DNA damage protection strategy in plants. Nat. Commun. 16, 60733. doi: 10.1038/s41467-025-60733-1. PMID: 40593573 PMC12215651

[B42] Szurman-Zubrzycka M. Jędrzejczyk P. Szarejko I. (2023). How do plants cope with DNA damage? A concise review on the DDR pathway in plants. Int. J. Mol. Sci. 24, 15895. doi: 10.3390/ijms241915895. PMID: 36768727 PMC9916837

[B43] Takahashi N. Ogita N. Takahashi T. Taniguchi S. Tanaka M. Seki M. . (2019). A regulatory module controlling stress-induced cell cycle arrest in Arabidopsis. eLife 8, e43944. doi: 10.7554/eLife.43944. PMID: 30944065 PMC6449083

[B44] Terwilliger T. C. Liebschner D. Croll T. I. Williams C. J. McCoy A. J. Poon B. K. . (2024). AlphaFold predictions are valuable hypotheses and accelerate but do not replace experimental structure determination. Nat. Methods 21, 110–116. doi: 10.1038/s41592-023-02087-4. PMID: 38036854 PMC10776388

[B45] Tomimoto S. Satake A. (2023). Modelling somatic mutation accumulation and expansion in a long-lived tree with hierarchical modular architecture. J. Theor. Biol. 565, 111465. doi: 10.1016/j.jtbi.2023.111465. PMID: 36931388

[B46] Tomimoto S. Satake A. (2024). Tropical trees inherit low-frequency somatic mutations. Trends Genet. 40, 636–637. doi: 10.1016/j.tig.2024.05.003. PMID: 39013722

[B47] Verwilt J. Mestdagh P. Vandesompele J. (2023). Artifacts and biases of the reverse transcription reaction in RNA sequencing. RNA 29, 889–897. doi: 10.1261/rna.079623.123. PMID: 36990512 PMC10275267

[B48] Virtanen P. Gommers R. Oliphant T. E. Haberland M. Reddy T. Cournapeau D. . (2020). SciPy 1.0: fundamental algorithms for scientific computing in Python. Nat. Methods 17, 261–272. doi: 10.1038/s41592-019-0686-2. PMID: 32015543 PMC7056644

[B49] Waneka G. Pate B. Monroe J. G. Sloan D. B. (2024). Exploring the relationship between gene expression and low-frequency somatic mutations in Arabidopsis with duplex sequencing. Genome Biol. Evol. 16, evae213. doi: 10.1093/gbe/evae213. PMID: 39365161 PMC11489876

[B50] Wang L. Ji Y. Hu Y. Hu H. Jia X. Jiang M. . (2019). The architecture of intra-organism mutation rate variation in plants. PloS Biol. 17, e3000191. doi: 10.1371/journal.pbio.3000191. PMID: 30964866 PMC6456163

[B51] Xian W. Carbonell-Bejerano P. Rabanal F. A. Bezrukov I. Reymond P. Weigel D. (2025). Minimizing detection bias of somatic mutations in a highly heterozygous oak genome. G3 (Bethesda) 15, jkaf143. doi: 10.1093/g3journal/jkaf143. PMID: 40543050 PMC12341919

[B52] Yu C. Hou L. Huang Y. Cui X. Xu S. Wang L. . (2023a). The multi-BRCT domain protein DDRM2 promotes the recruitment of RAD51 to DNA damage sites to facilitate homologous recombination. New Phytol. 238, 1073–1084. doi: 10.1111/nph.18787. PMID: 36727295

[B53] Yu H. Ma L. Zhao Y. Naren G. Wu H. Sun Y. . (2023b). Intraorganismal genetic heterogeneity in Leymus chinensis revealed by leaf and seed sequencing. Front. Plant Sci. 14. doi: 10.3389/fpls.2023.1157145. PMID: 37346123 PMC10280068

[B54] Zhang H. Gong Z. Zhu J. K. (2022). Active DNA demethylation in plants: 20 years of discovery and beyond. J. Integr. Plant Biol. 64, 2217–2239. doi: 10.1111/jipb.13423. PMID: 36478523

