# Supplementary material for: Transcriptome analysis reveals DNA repair–related clues associated with divergent leaf nuclear DNA diversity in Leymus chinensis
Source: Front Plant Sci. 2026 Apr 10;17:1804463. doi: 10.3389/fpls.2026.1804463 (PMC13106374; doi:10.3389/fpls.2026.1804463)
Supplement: Supplementary Figure 1 — Alignment overview and PCR validation for three nuclear loci used in clone-based variation analysis. (A) Multiple sequence alignment snapshots of clone-derived amplicons at three nuclear loci (MCM7, PsaE, and PsaL) in LC-ZK2 and LC-W. Each locus includes three biological replicates per material (labeled 1–3). (B) Representative agarose gel images showing successful PCR amplification of MCM7, PsaE, and PsaL in LC-ZK2 and LC-W; M, DNA ladder; [file DataSheet1.pdf]

Table S1. Summary statistics of RNA-seq data for *Leymus chinensis* leaf samples.

sample	Raw reads	Clean reads	Clean bases	Q20(%) (%)	Q30(%) (%)	GC Content(%)
WT1	53538376	52562586	7.86 Gb	97.92	94.01	53.27
WT2	48846496	48270634	7.10 Gb	97.44	95.41	54.84
WT3	46409818	45863038	6.87 Gb	98.11	94.5	55.12
ZK1	53399722	52846850	7.91 Gb	98.33	95.07	54.45
ZK2	50665518	49921498	7.44 Gb	98.27	94.98	54.78
ZK3	50299876	49664018	7.40 Gb	98.17	94.61	54.45

Note: 1 Gb = 1×10^9 bp

Table S2. Assembly metrics for the de novo transcriptome of *Leymus chinensis*.

Type	Unigene	Transcript
Total number	158997	280166
Largest length (bp)	11730	11730
Smallest length (bp)	201	201
Average length (bp)	690.46	847.07
N50 length (bp)	1027	1313
E90N50 length (bp)	2138	1855
BUSCO score	C:72.5%[S:70.7%;D:1.8%]	C:84.3%[S:21.3%;D:63.0%]

Note: BUSCO score: The assembly completeness was evaluated using BUSCO; a higher score indicates better completeness. “C” stands for Complete (values >70% are considered acceptable). Within C, “S” denotes Single-copy, meaning a gene in the BUSCO database is matched by a single sequence, while “D” denotes Duplicated, meaning a gene is matched by multiple sequences.

Table S3. Effects of *XRCC1* BRCT-domain nucleotide substitutions on codons and amino acids.

Site	1	2	3-5	6	7	8
Codons of	GGC	CCC	TCG	GAG	CAG	ATT
cDNA	GGT	TCC	GAT	GAT	AAG	GTT
Amino acid	G/S	P/S	S/D	E/D	K/Q	I/V

Note: The amino-acid haplotype h4 was used as the reference sequence. For each site, the upper row indicates the reference (codon/amino acid) and the lower row indicates the mutant state. Site numbering follows the cDNA alignment coordinates within the amplified XRCC1 BRCT-domain fragment. Nucleotide substitutions and the corresponding amino-acid changes are highlighted in red (synonymous substitutions are indicated by nucleotide changes without amino-acid replacement).

Table S4. Primers used for PCR validation of genes in *Leymus chinensis*.

**Unigene ID**	**Primer Direction (5'→3')**	**Primer Sequence**	**Primer Length / bp**	**Product Length / bp**
Actin	Forward	ATTGTGCTCAGTGGTGGGTCA	21	106
	Reverse	CCAATCCAAACACTGTACTTCCTC	24	
XRCC1	Forward	GATGGGGTGGTCTTTGTGCT	20	201
	Reverse	TGAGATCCATTCCTTGGCGAC	21	
PARP1	Forward	AAAAACAGCTGCTGGGCTTG	20	209
	Reverse	TCGTTGAAAATGTTAGGAGTTTCA	24	
FPG	Forward	CTTGGACAGTCGCCCTTGAT	20	202
	Reverse	TTGAACACTAGCAGCTTTATCTCCT	25	
OGG1	Forward	TCTCCCCGCTCCGATTCA	18	201
	Reverse	CGGTGAACTGGACCCAGAG	20	
APE1L	Forward	GGATGAAGAGCGACTGGTCC	20	220
	Reverse	GACCACCAGACACGGTAGTC	20	
LIG1	Forward	GGTCTGTACTTTTGGCTTTGATAT	24	206
	Reverse	TCAACCCTTCACAACTGGAGT	21	
PasE	Forward	ATGGCAAGCACCAACATGGCGT	22	646
	Reverse	TTAAGCAGCAACCTCCTTGATCTCG	25	
PsaL	Forward	ATGGCTACTGCATATGCTCCTC	22	741
	Reverse	GAAGAAGTATGGGAGGTCGAGC	22	
MCM7	Forward	GAGCAGGAACAAATTGACCGATTGG	25	1129
	Reverse	GGTGTACATGAACAACATGTCTTGCC	26	
XRCC1	Forward	GATGGGGTGGTCTTTGTGCTG	21	240
BRCT	Reverse	CCAGCATGCATAAGGTAAGGCTC	23	

Table S5. Corrected P values of the GO terms shown in Figure 3.

GO ID	Term Type	Description	P-value
GO:0006807	BP	nitrogen compound metabolic process	0.0000000747
GO:0044237	BP	cellular metabolic process	0.000000335
GO:0008150	BP	biological_process	0.013215476
GO:1901360	BP	organic cyclic compound metabolic process	0.000000193
GO:0006259	BP	DNA metabolic process	0.000000159
GO:0044260	BP	cellular macromolecule metabolic process	0.000000105
GO:0006974	BP	cellular response to DNA damage stimulus	0.000000292
GO:0006281	BP	DNA repair	0.000000173
GO:0006302	BP	double-strand break repair	0.0000000247
GO:0006310	BP	DNA recombination	0.0000000376
GO:0071103	BP	DNA conformation change	0.000000119
GO:0000724	BP	double-strand break repair via homologous recombination	0.000000249
GO:0006260	BP	DNA replication	0.0000000205
GO:0006268	BP	DNA unwinding involved in DNA replication	0.000000366
GO:0006270	BP	DNA replication initiation	0.000000617
GO:0043226	CC	organelle	0.0000926
GO:0043229	CC	intracellular organelle	0.000092
GO:0005634	CC	nucleus	0.00000421
GO:0043227	CC	membrane-bounded organelle	0.000832465
GO:0043231	CC	intracellular membrane-bounded organelle	0.00082783
GO:0032991	CC	protein-containing complex	0.002865417
GO:0042555	CC	MCM complex	0.000000231
GO:0097159	MF	organic cyclic compound binding	0.00000955
GO:0003676	MF	nucleic acid binding	0.000000124
GO:1901363	MF	heterocyclic compound binding	0.00000938
GO:0005488	MF	binding	0.002370799
GO:0140640	MF	catalytic activity, acting on a nucleic acid	0.0000000948
GO:0003824	MF	catalytic activity	0.040194381
GO:0140097	MF	catalytic activity, acting on DNA	0.000000393
GO:0003677	MF	DNA binding	0.000000198
GO:0016787	MF	hydrolase activity	0.0000096
GO:0036094	MF	small molecule binding	0.004009181

Note: Only the GO terms displayed in Figure 3 are listed here. Enrichment significance was corrected for multiple testing using FDR.

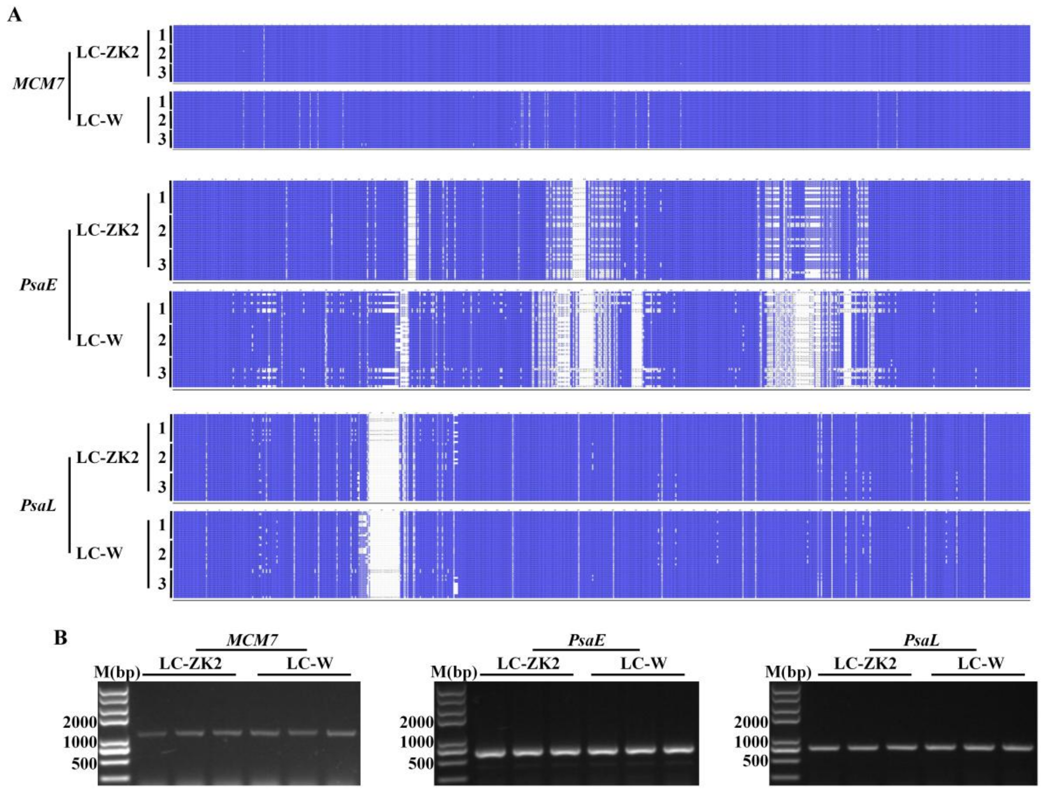

Figure S1. Alignment overview and PCR validation for three nuclear loci used in clone-based variation analysis.

(A) Multiple sequence alignment snapshots of clone-derived amplicons at three nuclear loci (*MCM7*, *PsaE*, and *PsaL*) in LC-ZK2 and LC-W. Each locus includes three biological replicates per material (labeled 1–3).

(B) Representative agarose gel images showing successful PCR amplification of *MCM7*, *PsaE*, and *PsaL* in LC-ZK2 and LC-W; M, DNA ladder.

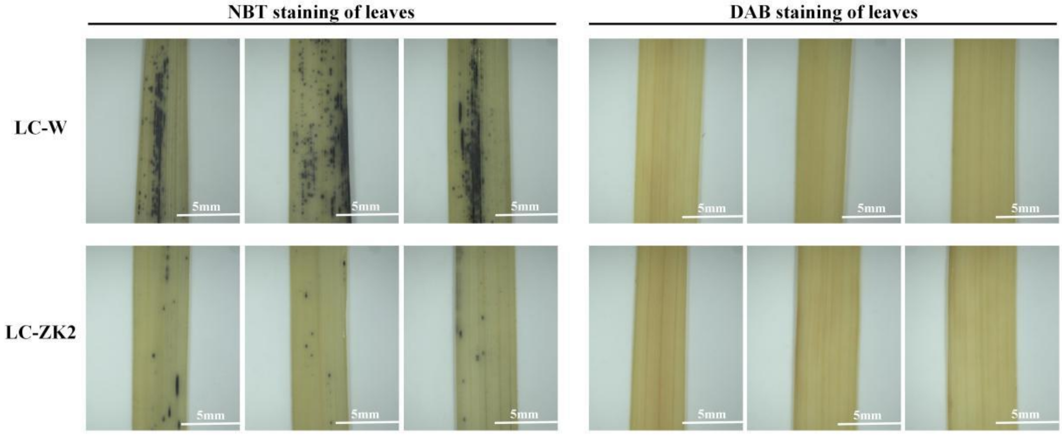

Figure S2. NBT and DAB staining of leaves from LC-ZK2 and LC-W.

NBT staining was used to detect superoxide anion (O_2^-), and DAB staining was used to detect hydrogen peroxide (H_2O_2). Representative leaf images are shown for three biological replicates of each material. NBT staining was stronger in LC-W than in LC-ZK2, whereas no obvious punctate brown signals were observed in the DAB-stained leaves of either material under the present conditions. Scale bars = 5 mm.

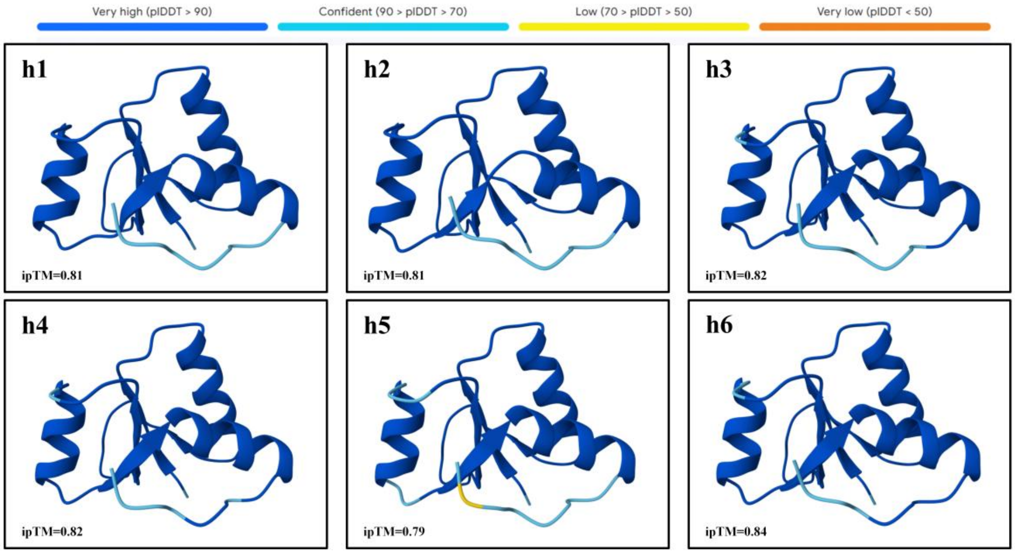

Figure S3. AlphaFold2-predicted structures of the XRCC1 BRCT domain for six amino-acid haplotypes.

Predicted BRCT-domain structures for six amino-acid haplotypes (h1 – h6) are shown in cartoon representation. Models are colored by per-residue confidence (pLDDT; dark blue, >90; blue, 70 – 90; light yellow, 50 – 70; orange, <50). The overall model score (ipTM) is indicated in each panel.
